# Free-Flowing Polymer-Bonded Powder Composition of Hexahydro-1,3,5-trinitro-1,3,5-triazine Using Solvent–Slurry Coating

**DOI:** 10.3390/polym16060841

**Published:** 2024-03-19

**Authors:** Muhammad Soulaman Khan, Muhammad Ahsan, Sarah Farrukh, Erum Pervaiz, Abdul Qadeer Malik

**Affiliations:** School of Chemical and Materials Engineering, National University of Sciences and Technology, Islamabad 44000, Pakistan; msoulaman.phdscme@student.nust.edu.pk (M.S.K.);

**Keywords:** coating of hexahydro-1,3,5-trinitro-1,3,5-triazine, matrix form of polyisobutylene polymer, flowability of the powder composition

## Abstract

A number of coating techniques have been used to improve the processability of high explosives. These techniques are typically used for developing compositions, such as boosters and fillers. The most typically used technique is the “solvent–slurry coating”. Several compositions of polymer-bonded explosives have been industrialized using this technique. The NUPC-6 polymer-bonded powder composition of hexahydro-1,3,5-trinitro-1,3,5-triazine is optimized using the solvent–slurry coating. It involved multiple processes, i.e., preparing a slurry of high explosives in an aqueous phase, dissolving the modified polymer binder in an organic solvent, maintaining both the solvent and slurry at controlled temperatures, introducing polymer binder solution and ingredients in the slurry, distilling the solvent, mixing contents homogeneously, filtering the polymer-coated hexahydro-1,3,5-trinitro-1,3,5-triazine composition, and drying in a vacuum oven. The phlegmatizing and hydrophobic agents enhance flowability and hydrophobicity. The mass flow rate, bulk density, tapped density, compressibility index, and Hausner ratio are determined to evaluate its flowability during filling operations. The results show that the composition is flowable using a filling funnel, with a 150 mm upper diameter, 25 mm flow diameter, and 136 mm total funnel height. The raw polymer binder was modified using diisooctylsebacate and SAE-10 oil. The additives in the composition enhance its flowability, and it might be used in underwater applications.

## 1. Introduction

There are different coating techniques, i.e., conventional and modern, for enhancing the performance and handling of high explosives. Recently, interest has risen in nanotechnology to create “reactive materials”, “metastable intermolecular composites”, or “pyrolants”. They are prominent in terms of high heat release per unit mass of substance, as they are nanoscale. However, their filling and extrusion technologies should also differ from conventional ones. Conventional filling and extrusion technologies also suffer from imperfections, i.e., resulting products may contain pores and cracks, and some technological stages are unsafe. Therefore, coating is one of the most typical conventional techniques for enhancing the performance and handling of high explosives. This technique uses different coating agents and coating methods [1,2]. The main coating methods used are “solvent–slurry coating”, “smeared wax coating,” and “aqueous slurry coating” using water-based polymers or wax emulsions [3]. These coating methods have been used and modified by different researchers. These are introduced one by one briefly hereafter.

Jingyu Wang coated cyclotetramethylenetetranitramine particles with new carbon material graphene oxide and Viton via a solvent–slurry coating [4]. Samson S. Samudre et al. worked on high explosive compositions coated with a solvent–slurry coating [5]. Hildebrant et al. performed wax coating onto the face of hexahydro-1,3,5-trinitro-1,3,5-triazine (generally known as RDX or Research and Development Explosive) particles using dispersing agent and water-based wax for coating RDX [6]. E. Gardner et al. introduced a wax coating of RDX slurry in a denser solvent than water [7]. Vernon D. Ringbloom performed coating crystalline explosives with polyethylene wax emulsion [8]. The aqueous slurry coating was used for automatic pelleting without needing other solvents for RDX and polyethylene wax, concentrating on the 0.5–5% wax range [9]. Richard H. Stresau et al. performed work on the aqueous slurry coating process and calcium stearate coating onto the face of ultra-fine particles of RDX [10]. The polymer adsorbs to the surface of explosive particles [11]. The energetic powder materials may also be coated using mineral jelly and similar materials to give a practical high explosive [12]. The aqueous polyurethane dispersions were also evaluated for use in high-explosive molding powders [13]. Ian J. Dagley et al. developed booster compositions from RDX via a coagulation technique using aqueous polyurethane, acrylic, ethylene–vinyl acetate dispersions, and the additional coating of zinc stearate [14]. Friedrich-Ulf Deisenroth applied an aqueous polyurethane binder in dispersion for high explosive compositions [15]. Ya-Jun Zhang et al. introduced the idea that modifying the surface properties of high explosives has theoretical and practical significance in applications [16]. Michal and Carolina worked on modifying explosives in an aqueous suspension using phlegmatizing agents [17]. Lemi Türker and Serhat Varis structurally modified RDX by making some slight molecular-level modifications in the explosive structure [18]. Hongyan Sun et al. modified the surface properties of high explosives using spray-drying [19]. Philip Kneisl and Karri A. Brock modified the surface of RDX by recrystallization of RDX using two solvents to increase the sphericity of RDX crystals for reducing sensitivity [20]. Xiang Yan et al. modified RDX by suspension spray technology using Estane 5703 as a binder [21]. Xue-Xue Zhang et al. modified high explosives by crystallization for particle size and morphology tuning [22]. Amiya Kumar Nandi et al. modified cyclotetramethylenetetranitramine with 1,3,5-Triamino-2,4,6-trinitrobenzene [23]. Xiang Yan et al. designed a device to evaluate binder characteristics for high explosives [24]. Lu Zhang et al. modified membranes with diisooctylsebacate as an excellent antibacterial agent [25]. Hulin Wu et al. reduced the sensitivity of RDX using polyvinylidene fluoride as an energetic binder [26]. Jiahao Liang et al. analyzed the interactions of hydroxyl-terminated polybutadiene and hydroxyl-terminated block copolyether prepolymer with ammonium perchlorate [27]. C. W. An et al. used hydroxyl-terminated polybutadiene, isophorone diisocyanate, and 2,4,6-trinitrotoluene to coat RDX particles via solvent evaporation and aqueous slurry melting [28].

Worldwide research has been performed to develop different high-explosive molding powders. Good molding powders should have a high bulk density and should be flowable and dustless [29,30]. Some of the important compositions used by RDX are composition A-3, composition A-4, composition A-6, composition B, composition CXM-3, composition CXM-6, composition CXM-7, and composition CXM-8. These are used as boosters and fillers. Other explosives like cyclotetramethylenetetranitramine are also used in different compositions. The composition PBXN-5 contains cyclotetramethylenetetranitramine and a copolymer [31]. L. D. Hampton used the solvent–slurry coating to develop the composition based on RDX, known as CH-6 composition [32]. It is used as a booster and filler.

The trends of additive manufacturing and advances in energetic materials are based on the synthesis of novel constituents and modification of the current formulations. The latter trend comprises various methods [33]. The research work in this article aims to develop a new polymer-bonded powder composition of RDX, based on the solvent–slurry coating by introducing plasticizer and lubricant in polymer binder and process optimization, applying both the trends on composition CH-6. The research methodology covers the selection of suitable solvent, gradation of RDX, modification of raw polymer with a high weight average molecular mass, precipitation of calcium stearate, and addition of flow-enhancing agent graphite. The block flow diagram for the new polymer-bonded powder composition of RDX is established. The raw and modified materials have been characterized by studies, i.e., boiling ranges of solvents, the thermal stability of RDX, functional groups of the raw polymer and modified polymer, crystallite forms of used stearates and graphite, variation in roughness, thickness, and topography of thin films of raw and modified polymer, and the morphology of particles of RDX.

The developed polymer-bonded powder composition and RDX have been characterized by studies, i.e., shapes of particles, mass flow rate, bulk density, tapped density, compressibility index (CI), and Hausner ratio (HR). The results show that the new polymer-bonded composition is flowable based on results of bulk density, tapped density, average compressibility index, and average Hausner ratio. Its mass flow rate and bulk density were measured using a funnel having a 150 mm upper diameter, 25 mm flow diameter, and 136 mm total funnel height. Similar methods were used by Ekaterina K. Kosareva et al. [34]. Its tapped density was measured using a 50 mL measuring cylinder. Its velocity of detonation (VOD) is measured to compare it with 2,4,6-Trinitrophenylmethylnitramine, known as Tetryl. It might be used in boosters of large sizes and as filler. It is more energetic than Tetryl.

## 2. Materials and Methods

The raw materials used for this research work are polyisobutylene (Sigma-Aldrich, Burlington, MA, USA), diisooctylsebacate (locally available), SAE-10 grade oil (shell), graphite (BDH, AR Grade, Mumbai, India), sodium stearate (BDH, AR Grade), calcium chloride (BDH, AR Grade), n-octane (Merck, AR Grade, Rahway, NJ, USA), toluene (Merck, AR Grade), n-hexane (Merck, AR Grade), and RDX of Pakistan Ordnance Factories (POF, Punjab, Pakistan).

**Figure 1:**
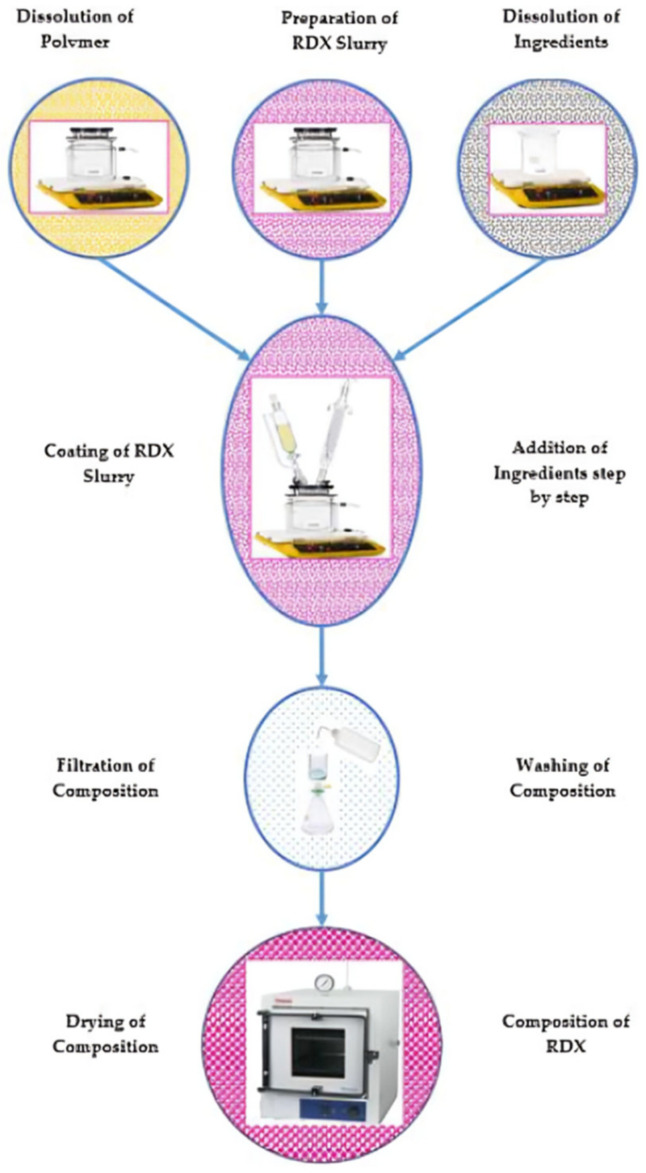
Schematic diagram of the solvent–slurry coating process for composition of RDX.

The labware used for the polymer binder solution in solvent and the RDX–water slurry includes a hot plate, magnetic stirrer, and sealed jacketed glass vessel. The labware used for coating polymer binder on the RDX–water slurry includes a hot plate, magnetic stirrer, sealed jacketed glass vessel, constant pressure funnel, and condenser. The labware used for the preparation of aqueous solutions of sodium stearate, calcium chloride, and aqueous slurry of graphite includes hot plates, magnetic stirrers, and glass beakers. These solutions and slurry were fed in batches using a constant pressure funnel. The composition is filtered, washed using a distilled water wash bottle, and dried in the composition under vacuum. The different steps are shown schematically in Figure 1.

The research was performed on the solvent–slurry coating to develop a flowable, polymer-bonded powder composition of RDX. This technique involved following stepwise processes. In the first step, the raw or modified polyisobutylene polymer was dissolved in the solvent and heated up to 45 °C using a jacketed glass vessel of 1 L, polytetrafluoroethylene coated magnetic stirrer, and flame-proof hot plate. In a second step, a 10% (*w*/*w*) RDX–water slurry was prepared in a jacketed glass vessel of 1 L with a polytetrafluoroethylene-coated magnetic stirrer and flame-proof hot plate. It was heated up to 50 °C. In the third step, the solution of raw polyisobutylene polymer or modified polyisobutylene polymer was added to the RDX slurry in a dropwise manner using a constant pressure funnel, maintaining the temperature at 50 °C. In the fourth step, the solvent was distilled from the RDX slurry at the boiling range of the solvent, leaving a raw or modified polyisobutylene polymer coated onto the face of the particles of RDX using a glass condenser.

In the fifth step, 13% (*w*/*w*) sodium stearate solution was prepared in distilled water using a glass beaker with a magnetic stirrer on a hot plate and heated up to 50 °C. The sodium stearate solution was added to the coated hexahydro-1,3,5-trinitro-1,3,5-triazine slurry at 50 °C. The contents were stirred for 25 min to distribute them homogeneously. In the sixth step, calcium chloride was dissolved in a solution containing 20% (*w*/*w*) calcium chloride in distilled water using a glass beaker with a magnetic stirrer and hot plate. Calcium chloride solution was mixed with the contents at the end of the fifth step, maintained at 50 °C. The precipitation reaction of sodium stearate and calcium chloride solutions results in the introduction of calcium stearate as a dispersant in the composition. The whole mixture was maintained at 50 °C for 30 min while stirring. In the seventh step, the graphite was suspended in slurry form in distilled water using a glass beaker with a magnetic stirrer on a hot plate and heated up to 60 °C. The graphite slurry was added to the contents at the end of the sixth step and maintained at 50 °C. The contents were continuously stirred to distribute graphite as a grey color throughout the composition. In the eighth step, a composition containing coated grains of RDX, graphite, calcium stearate, and raw or modified polymer binder was formulated. This composition was filtered, cooled to room temperature, washed from alkali contents along NaCl with distilled water to neutral pH, and dried in a vacuum oven. This methodology was used for experimental work discussed hereafter.

The experimental work was performed to develop the composition of RDX using n-octane, toluene, and n-hexane as solvents for polymer binders. The boiling ranges of the solvents were 125–126 °C, 110–111 °C, and 68–69 °C, respectively. The n-hexane has the lowest processing temperature and toxicity of three of the solvents. It was selected for the preparation of a new flowable, plastic-bonded composition. The size range of RDX particles was graded for achieving homogeneously polymer-coated particles of RDX using a jacketed glass vessel, polytetrafluoroethylene coated magnetic stirrer, and a flame-proof hot plate. The finely recrystallized Class 1 RDX consisted of an extensive size range of up to −325 mesh. The experimental work for coating different-sized particles of RDX was performed. The microscopic views showed that the size range passed 100 mesh and retained 150 mesh, resulting in a good-quality polymer coating. It was selected as the optimized particle size range for developing the new composition. When finer sizes than 150 mesh were used for the composition preparation, it resulted in a non-homogeneous polymer binder distribution. The gradation of RDX was performed using a flame-proof Meinzer-II Sieve Shaker with flame-proof sieves of brass.

The raw polymer polyisobutylene with a weight of average molecular mass, M_w_~4,200,000 gm/mol, and number average molecular mass, M_n_~3,100,000 gm/mol was used as raw polymer binder for preparing the flowable, polymer-bonded powder composition of RDX. It took too much time for complete dissolution in n-hexane, i.e., 24 h at 50 °C in n-hexane. The tendency to form large-sized drops while pouring the dissolved raw polymer polyisobutylene in n-hexane was observed. It was a problem in achieving thin-film polymer coating onto the face of particles of RDX. Moreover, the powder composition prepared by coating raw polymer polyisobutylene was observed to have roughness, hardness, and less plasticity. The plastic nature is required for the extrusion of large boosters and linear-shaped charges. Therefore, the raw polymer polyisobutylene was modified to reduce these shortcomings by adding the highly polar diisooctylsebacate and lubricant SAE-10 oil. The mixing setup used for modification of the polymer binder was a 1-L jacketed glass vessel, polytetrafluoroethylene coated magnetic stirrer, and thermolyne flame-proof hot plate. The raw polymer binder was dissolved in n-hexane at 45 °C by stirring for 24 h. The diisooctylsebacate and SAE-10 oil were added to the polymer binder solution. The solution was mixed homogeneously, and n-hexane was distilled using a condenser. It gives a gelled form of polymer matrix binder. The polymer matrix binder consists of ingredients: 33% polyisobutylene polymer, 53% diisooctylsebacate, and 14% SAE-10 oil.

A 10% RDX slurry containing 10% RDX and 90% water by weight was prepared in a jacketed glass vessel of 1-L capacity with a polytetrafluoroethylene-coated magnetic stirrer and flame-proof hotplate. The slurry was heated up to 50 °C. The modified polymer binder was dissolved in the n-hexane and heated up to 45 °C. The polymer binder solution was added dropwise in the hexahydro-1,3,5-trinitro-1,3,5-triazine slurry. After adding the polymer binder solution, the contents were mixed and heated on the hot plate for 15–30 min. The n-hexane was distilled at 68–69 °C using a circulated condenser. The hexahydro-1,3,5-trinitro-1,3,5-triazine particles are left with a coating of polymer matrix composition of polyisobutylene.

The additive calcium stearate in the mixing process was introduced during the precipitation reaction. It was used to control the particle size of RDX after distilling solvent. It acted as a dispersant. It avoided the agglomeration of coated particles with each other. The sodium stearate and calcium chloride solutions were prepared in distilled water in separate glass beakers. The sodium stearate solution was added to a slurry of polymer-coated RDX. The calcium chloride solution was added to the mixture of sodium stearate aqueous solution. The reaction of sodium stearate and calcium chloride resulted in the precipitation of calcium stearate as a dispersant homogenously throughout the composition. The chemical reaction is given as follows:

2C_18_H_35_COONa (aq) + CaCl_2_ (aq) → (C_18_H_35_COO)_2_Ca (s)+ 2NaCl (aq)

The additive graphite was added to the composition after precipitation and homogeneous distribution of dispersant, calcium stearate. Using an aqueous phase, the graphite was added to polymer-coated and dispersed hexahydro-1,3,5-trinitro-1,3,5-triazine particles. The composition was filtered and washed from alkali contents along NaCl with distilled water. The ingredients of the composition are 97.5% RDX, 0.17% polyisobutylene polymer, 0.26% diisooctylseba-cate, 0.07% SAE-10 oil, 1.5% calcium stearate, and 0.5% graphite.

**Figure 2:**
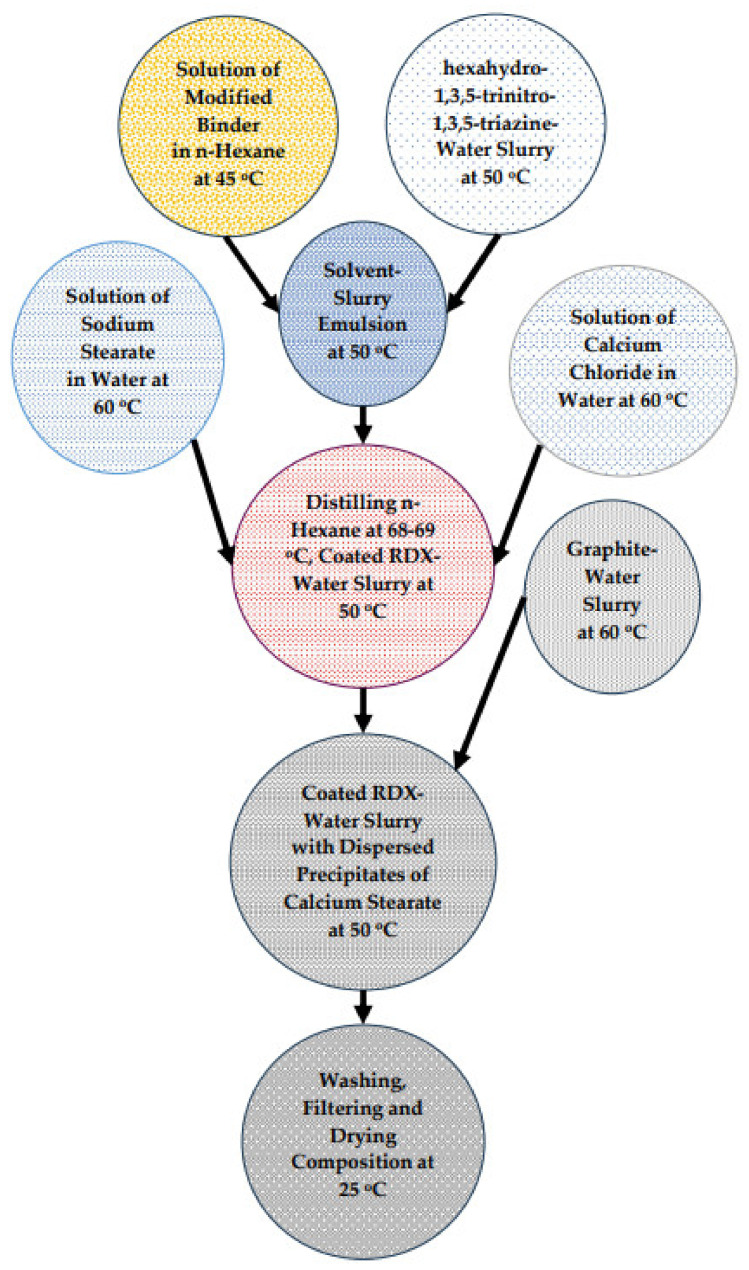
Block flow diagram of optimized process for NUPC-6 composition.

The flowable, plastic-bonded composition containing RDX, polyisobutylene polymer, diisooctylsebacate, SAE-10 oil, calcium stearate, and graphite was prepared. It was named NUPC-6 composition. The complete block flow diagram of the process for its preparation is given in Figure 2.

The solvents were characterized by the boiling point ranges for the selection of the solvent. The METTLER TOLEDO-DSC 1 differential scanning calorimeter was used to characterize the thermal stability and the melting point of hexahydro-1,3,5-trinitro-1,3,5-triazine. Differential scanning calorimetry (DSC) of two samples of RDX powder was performed. The nitrogen purging was 100 mL per minute during the DSC test. The samples were heated at ten °C per minute from 30 to 350 °C with masses taken 1.9 mg each in an open pan. The Perkin Elmer Spectrum 100 Fourier-Transform Infrared (FTIR) Spectrometer was used to identify the functional groups of raw polyisobutylene polymer and modified polyisobutylene polymer. The potassium bromide was used as an inert to infrared rays for pelleting samples for FTIR Testing.

**Figure 3:**
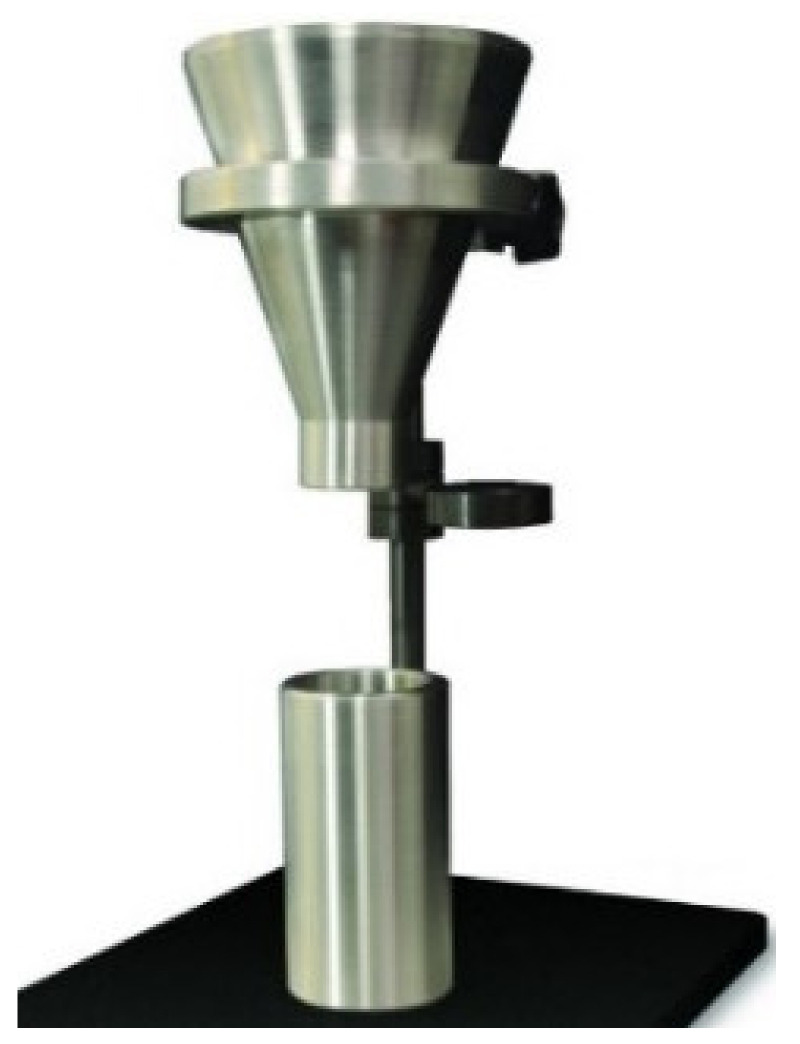
Mass flow rate measuring apparatus.

The BRUKER D2 Phaser x-ray diffractometer (XRD) (Billerica, MA, USA) was used for the characterization of crystalline forms. The sample of calcium stearate was prepared after an independent chemical reaction between solutions of sodium stearate and calcium chloride. The precipitates were washed to neutral pH from alkali. The crystallite forms of sodium stearate, calcium stearate, and graphite powders were characterized. The JEOL JSPM-5200 scanning probe microscope (Tokyo, Japan) or atomic force microscope (AFM) was used to characterize the thin films of raw polymer binder and modified polymer binder. Two solutions of raw polymer binder and modified polymer binder were prepared in n-hexane at 45–50 °C on the hot plate using a magnetic stirrer at 200 RPM for 2 h each. The solutions were sucked into the syringe. The syringe was bleeding. Two to three syringe drops were injected on glass slides to prepare samples for atomic force microscopy. The samples were dried and cured in the oven. The atomic force microscopy was performed to characterize the thin-film samples. The JEOL JSM-6490A analytical scanning electron microscope (SEM) was used to characterize the size range and morphology of the shape and surface of RDX. The OPTIKA B-600 MET microscope (Ponteranica, Italy) was used for the characterization of the shape of particles of hexahydro-1,3,5-trinitro-1,3,5-triazine, as well as their composition. The mass flow rates of RDX and new polymer-bonded powder composition were measured using a self-made apparatus as shown in Figure 3, containing a 60° inclined funnel (with a 150 mm upper diameter, 25 mm flow diameter, 106 mm height of inclined length, and 136 mm total funnel height), stopper, stand, receiver and a stopwatch. The mass flow rates were measured by taking known masses of samples in 60° inclined funnels and freely dropping them in the receiving cylinder. The dropping times were recorded using a stopwatch as flow time, and the mass per unit flow time was recorded as the mass flow rate of the sample.

**Figure 4:**
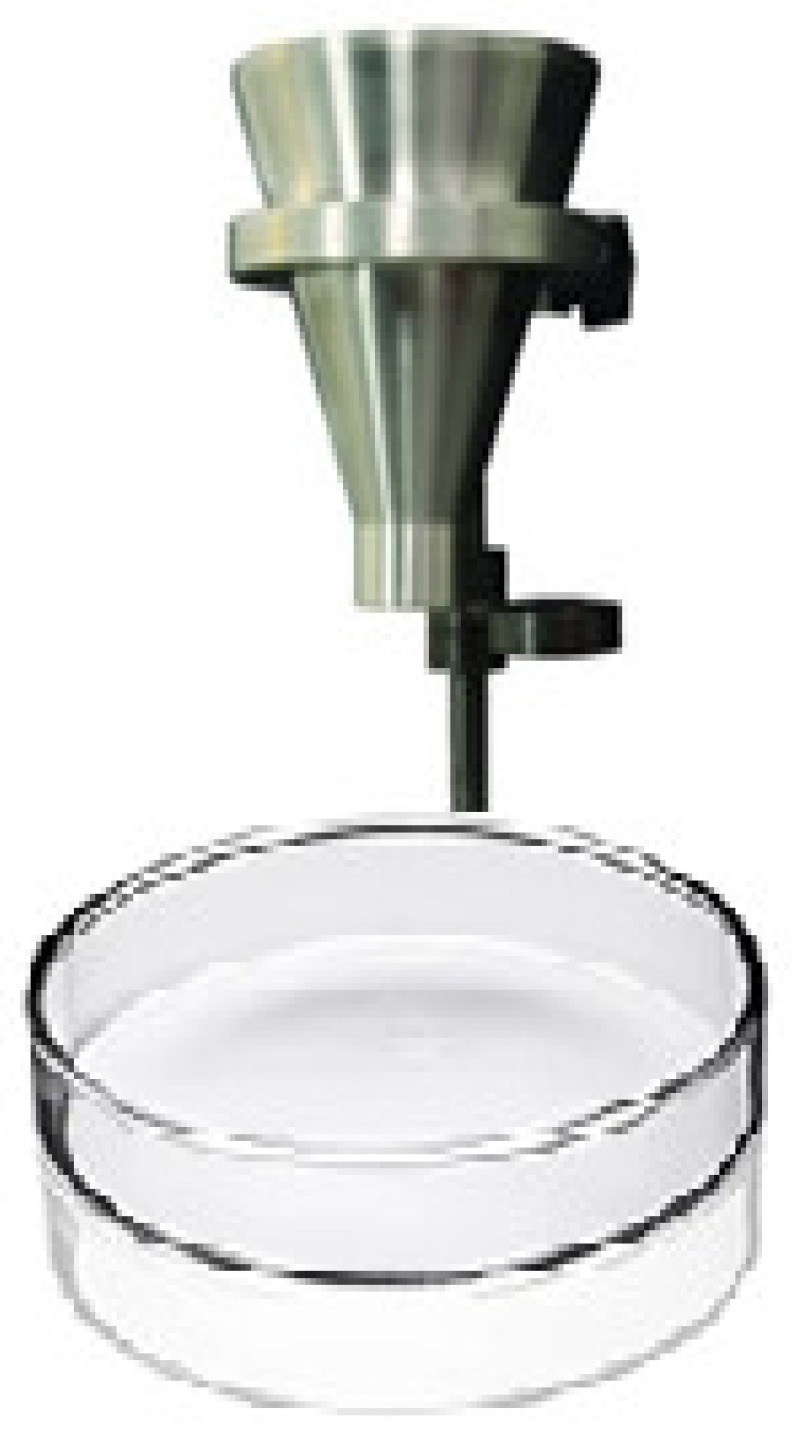
Bulk density measuring apparatus.

The compressibility index of RDX and new polymer-bonded powder composition were measured using experimental results of bulk densities and tapped densities. The bulk densities of RDX and new polymer-bonded powder composition were measured using self-made apparatus as shown in Figure 4, containing a 60° inclined funnel (with a 150 mm upper diameter, 25 mm flow diameter, 106 mm height of inclined length and 136 mm total funnel height), stopper, stand, China Petri dish, and a millimeter scale.

The bulk densities of the samples were experimentally measured by dropping the known masses through the funnel in a China Petri dish. The heights and diameters of heaps after dropping samples were determined manually. The volumes of heaps were determined using Equation (1) of the cone volume as given below.

(1) $$\mathrm{Heap}\;\mathrm{Volume}=\frac{1}{3}h\pi R^{2}$$

where ‘*R*’ is the radius and ‘*h*’ is the height of the heap of powder samples. The mass per unit heap volume was characterized to be the bulk density of samples. The tapped densities of RDX and newly developed, polymer-bonded powder composition were experimentally determined using 50 mL measuring cylinders. The known masses of the samples were tapped a hundred times gently in the measuring cylinders. The tapped densities of the samples were calculated by using the finally achieved tapped volumes. The tapped mass per unit tapped volume was characterized to be the tapped density of the sample. The compressibility index is a measure of the compressibility of the samples. The compressibility index of the powder samples is given in Equation (2) [35] as follows:

(2) $$\mathrm{Compressibility}\;\mathrm{Index}\;\mathrm{of}\;\mathrm{Powder}=\left(1-\frac{Bulk\;Density\;of\;Powder}{Tapped\;Density\;of\;Powder}\right)\times 100$$

The flow property of a booster powder is very important from its application point of view as a booster and filler. Its flowing property is highly desired for filling and extrusion operations. The Hausner ratio gives a measure of the flow property of the composition. The experimental results of the tapped density and the bulk density were used to calculate Hausner’s ratio of the RDX and the composition. The Hausner’s ratio of powders is given in Equation (3) [35] as follows:

(3) $$\mathrm{Hausner}’s\;\mathrm{Ratio}\;\mathrm{of}\;\mathrm{Powder}=\frac{Tapped\;Density\;of\;Powder}{Bulk\;Density\;of\;Powder}$$

**Figure 5:**
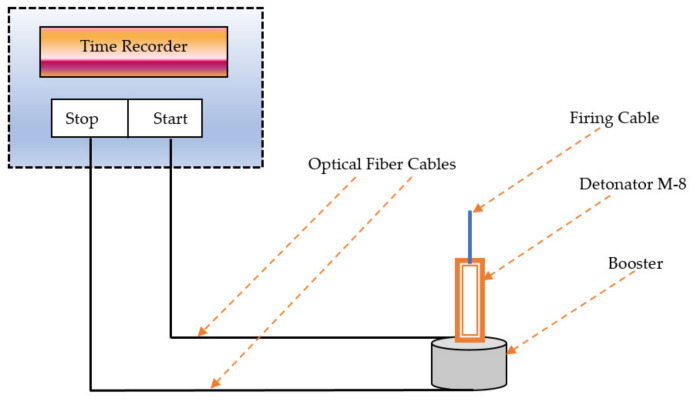
Schematic diagram of the fiber optic technique for VOD measurement.

The KONTINITRO EXPLOMET-FO-2000, a detonating velocity measuring system based on fiber optic technique, was used to measure the velocity of detonation (VOD) of the NUPC-6 composition and Tetryl. The schematic diagram of the fiber optic technique used for the VOD is shown in Figure 5.

The sensor of the first or the upper optical fiber cable was used to indicate the start signal of the detonation. The sensor of the second or lower optical fiber cable was used to indicate the stop signal of the detonation. The time duration was recorded by a time recorder built into the system. The system measured the VOD as the distance between sensor probes divided by the time recorded.

## 3. Results

### 3.1. Boiling Ranges of Solvents

**Figure 6:**
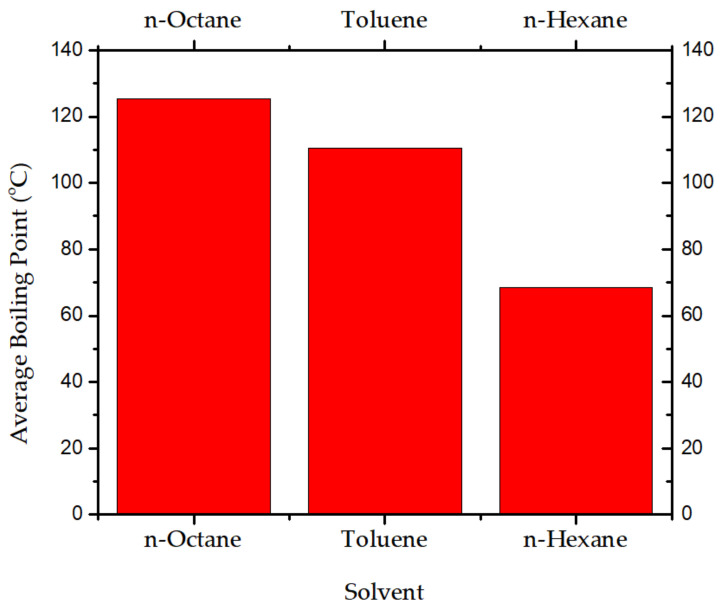
Average boiling temperatures of solvents (°C).

The composition of RDX was prepared using n-octane, toluene, and n-hexane as solvents. The boiling point ranges of n-octane, toluene, and n-hexane are 125–126 °C, 110–111 °C, and 68–69 °C, respectively. The results of the average boiling temperatures of the solvents are plotted in Figure 6.

### 3.2. Differential Scanning Calorimetry (DSC) of RDX

**Figure 7:**
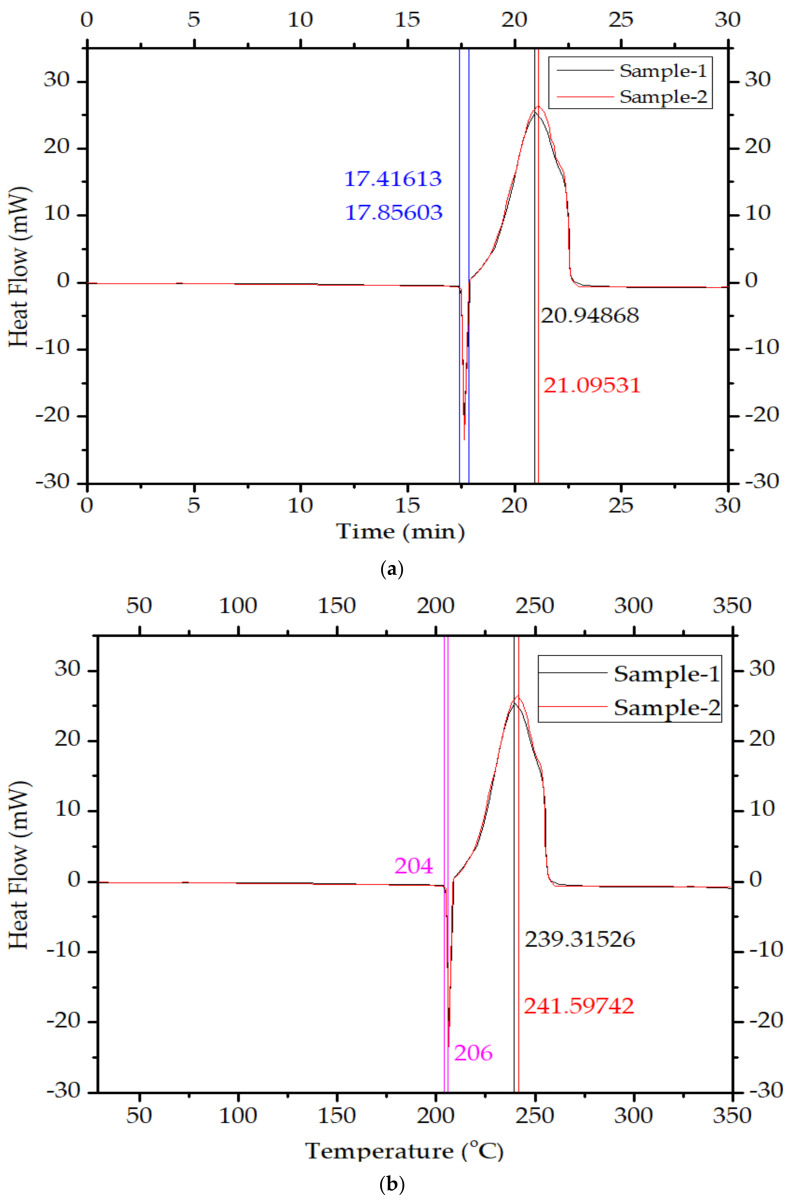
Results of METTLER TOLEDO-DSC 1 differential scanning calorimeter thermographs of RDX samples: (**a**) temperature vs. heat flow and time vs. heat flow of sample 1; (**b**) temperature vs. heat flow of sample 2 and time vs. heat flow of sample 2.

The DSC thermographs of two samples of RDX are shown in Figure 7.

### 3.3. Fourier-Transform Infrared (FTIR) Spectroscopy of Raw and Modified Polymer Binder

**Figure 8:**
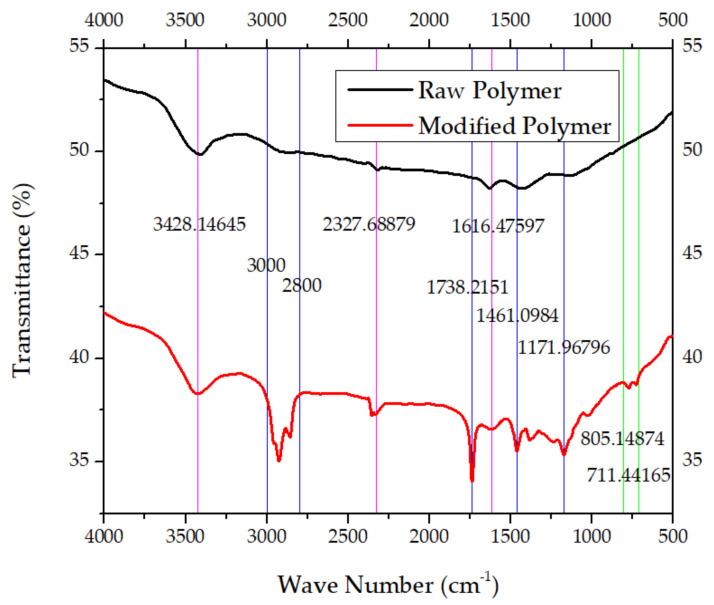
Results of FTIR of raw and modified polymer samples: wave number vs. percent transmittance of raw and modified polymer binder samples.

The FTIR results of functional groups in raw and modified polymer binders are given in Figure 8.

### 3.4. X-ray Diffraction (XRD) of Stearates and Graphite

**Figure 9:**
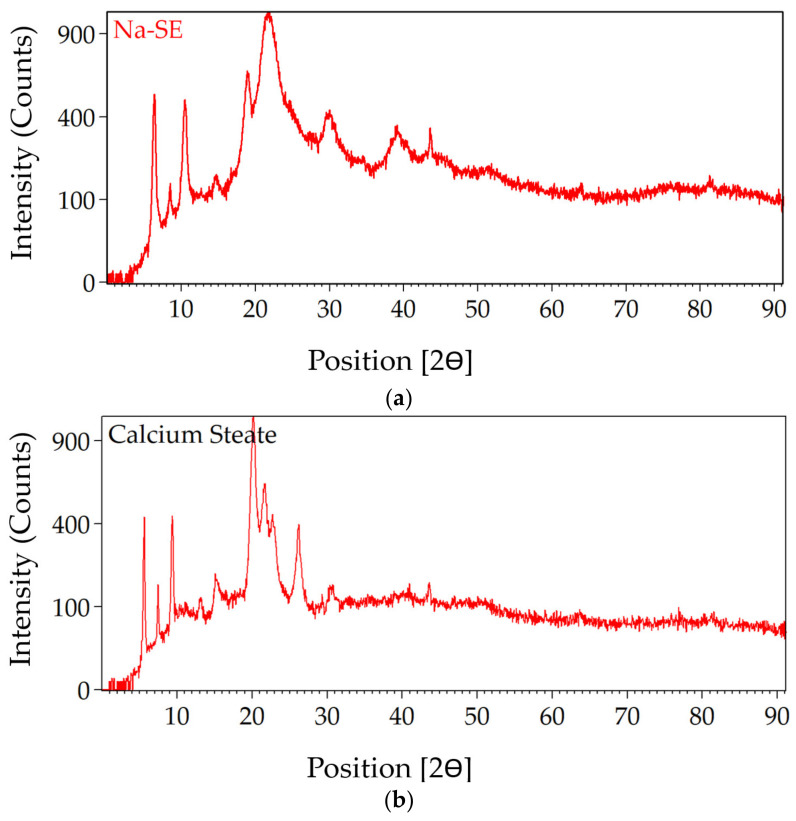

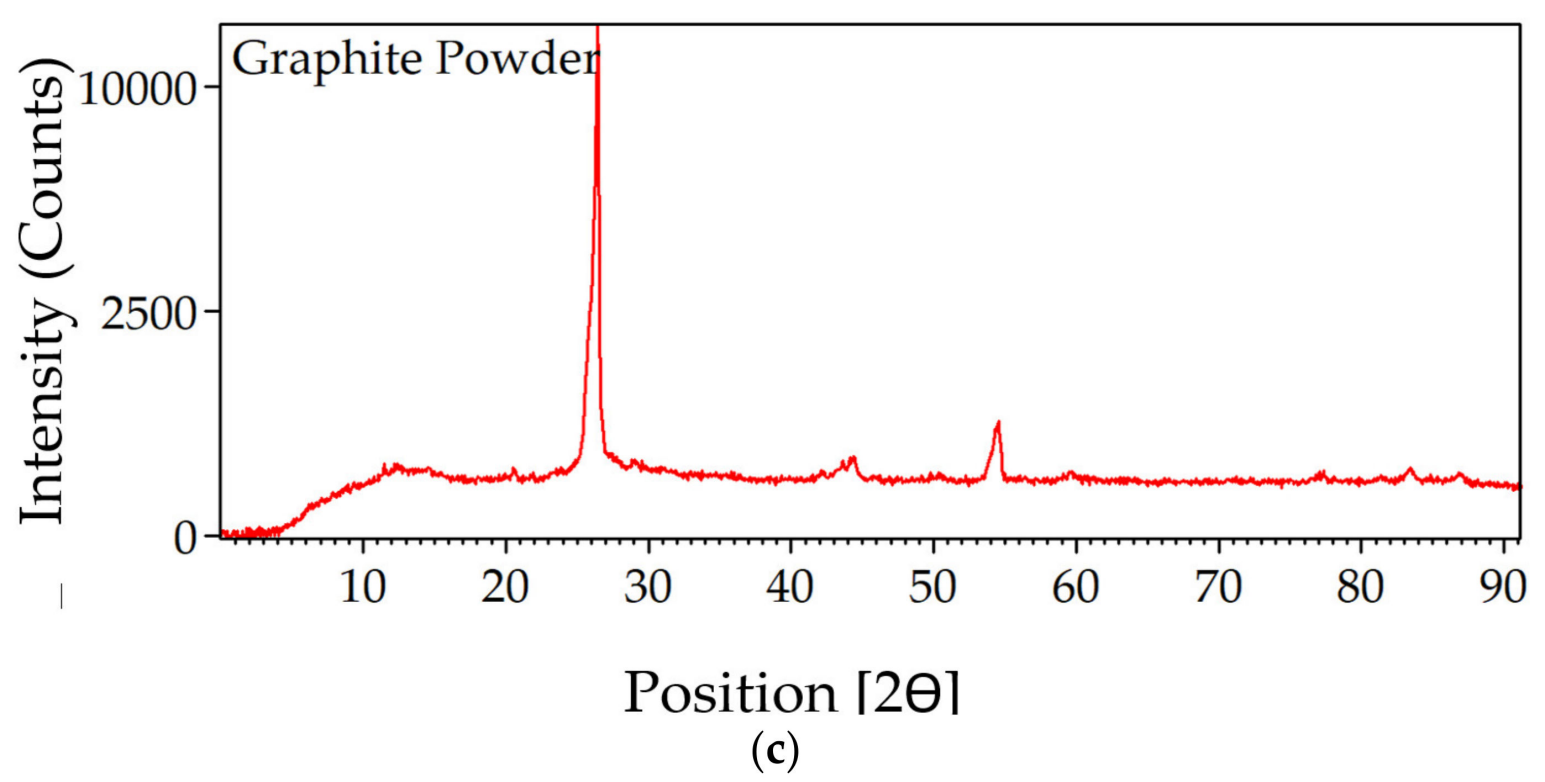
Results of XRDs: (**a**) XRD graph of plane angle vs. intensity in the count of sodium stearate; (**b**) XRD graph of plane angle vs. intensity in the count of calcium stearate; (**c**) XRD graph of plane angle vs. intensity in the count of graphite.

The results of the XRD pattern of sodium stearate, calcium stearate, and graphite are shown in Figure 9.

### 3.5. Atomic-Force Microscopy (AFM) of Thin-Films of Raw and Modified Polymer Binder

**Figure 10:**
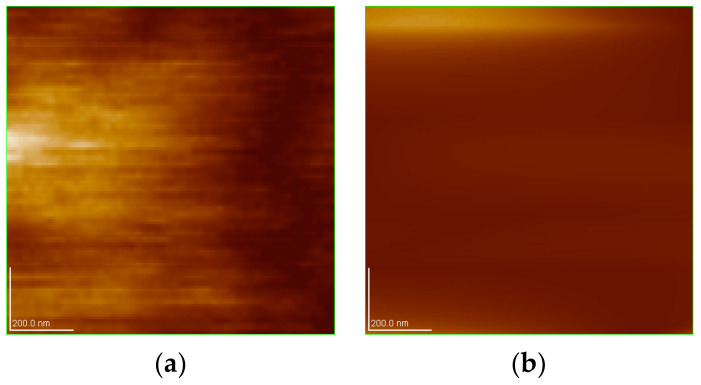
AFM of 200 nm × 200 nm and thin films of polymer binder samples: (**a**) thin film of raw polymer binder; (**b**) thin film of modified polymer binder.

**Figure 11:**
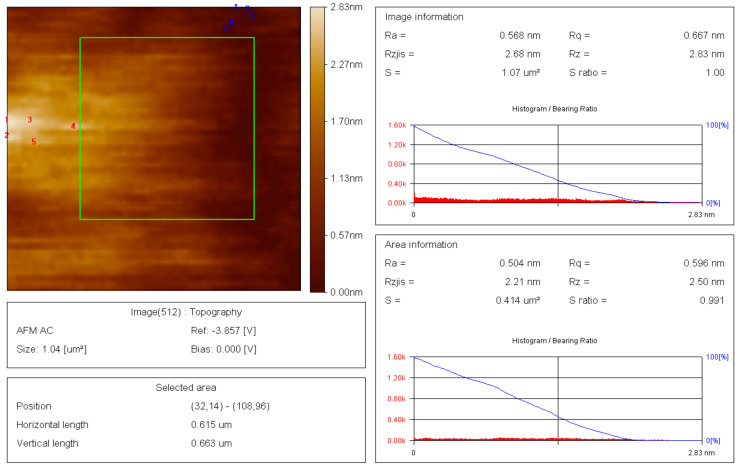
Roughness variation along the *Z*-axis of the raw polymer binder.

**Figure 12:**
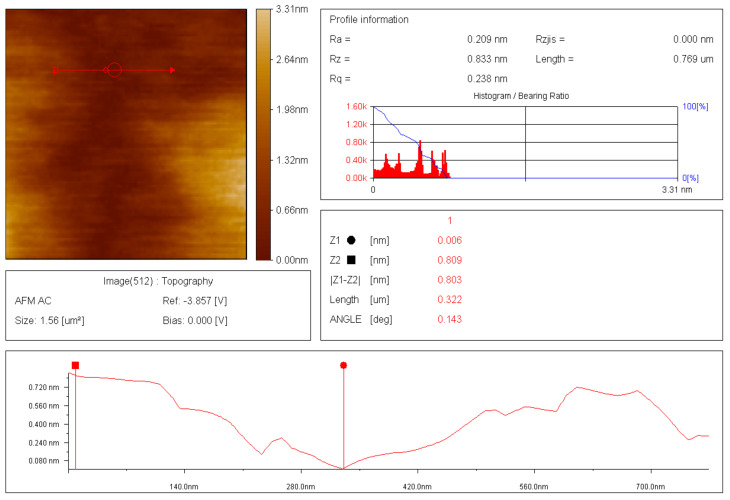
Two-dimensional thickness variation in thin film of raw polymer binder.

**Figure 13:**
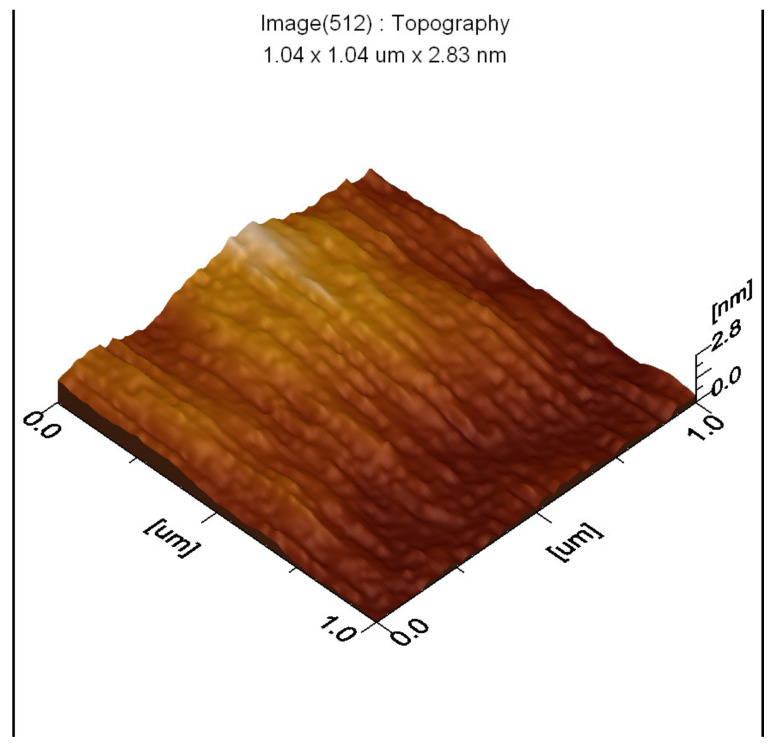
Topography image of thin film of raw polymer binder.

**Figure 14:**
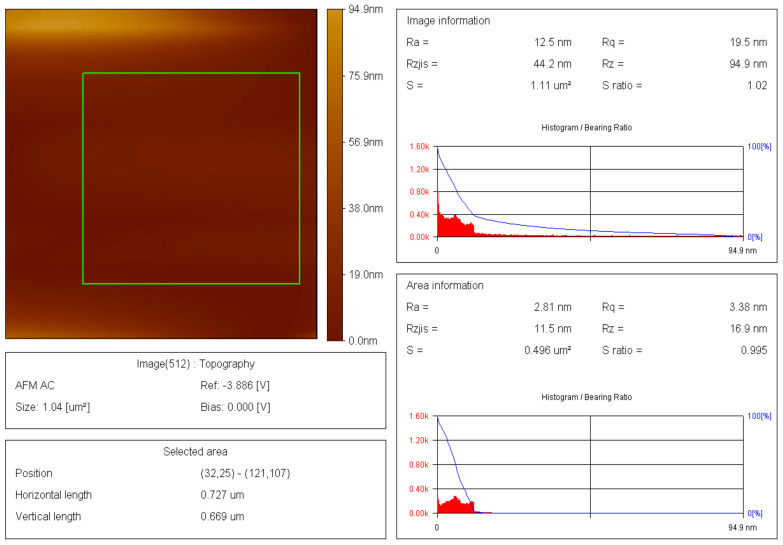
Roughness variation along the *Z*-axis of the modified polymer binder.

**Figure 15:**
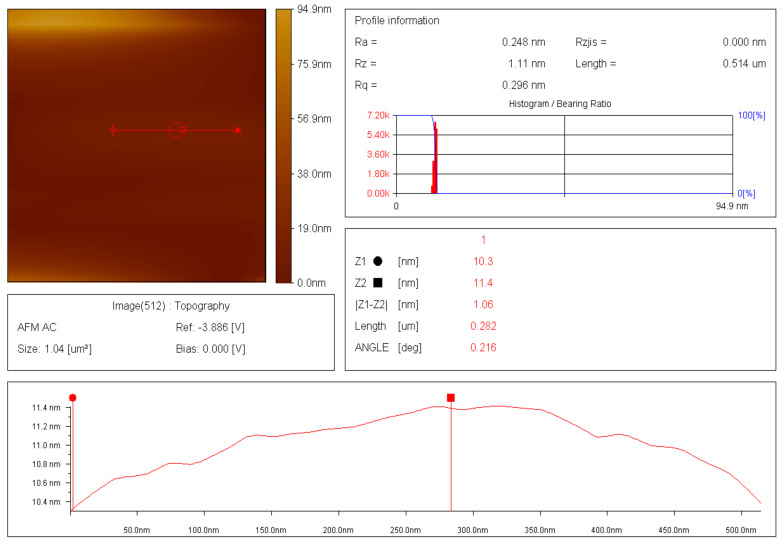
Two-dimensional thickness variation in thin film of modified polymer binder.

**Figure 16:**
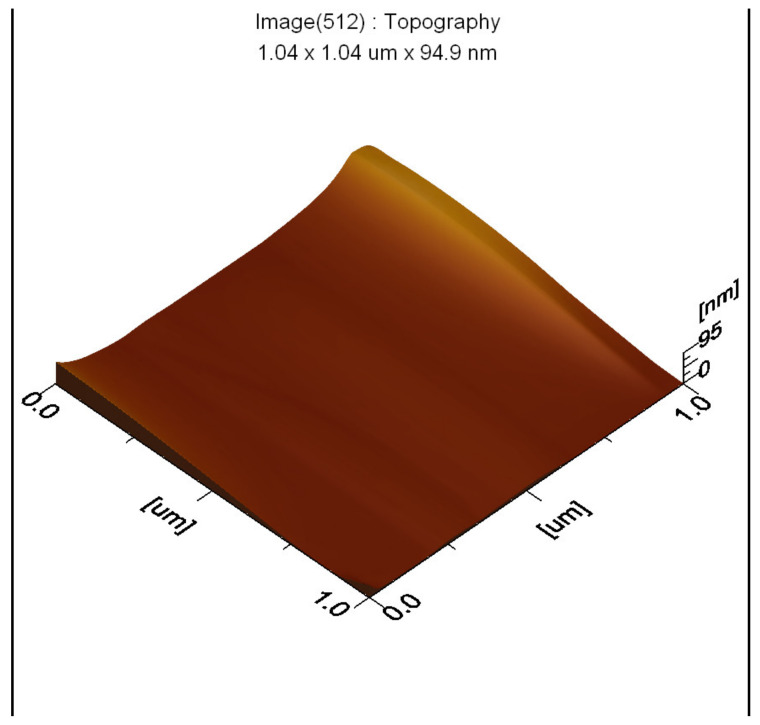
Topography image of thin film of modified polymer binder.

The thin films and results of AFM of 200 nm × 200 nm thin films of the raw and modified polymer binders are shown in Figure 10, Figure 11, Figure 12, Figure 13, Figure 14, Figure 15 and Figure 16.

### 3.6. Scanning Electron Microscopy (SEM) of RDX and Samples

**Figure 17:**
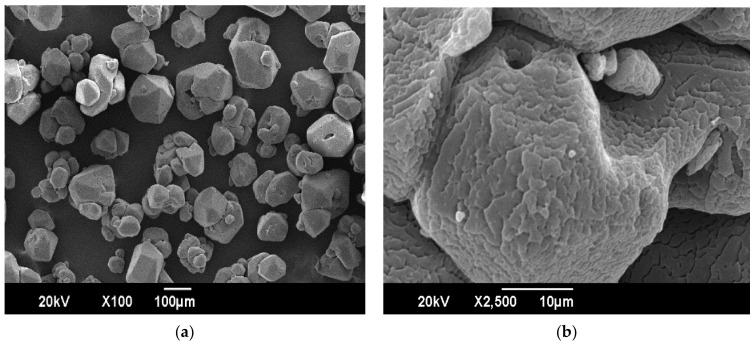
Views of SEM of RDX: (**a**) morphology of particles of RDX; (**b**) morphology of surface of RDX.

The results of the SEM of particles and the surface of the RDX sample are given in Figure 17. The cracks are apparent on the surface due to decomposition based on exposure to high voltages.

### 3.7. Metallurgical Microscopic Examination of RDX and NUPC-6 Samples

**Figure 18:**
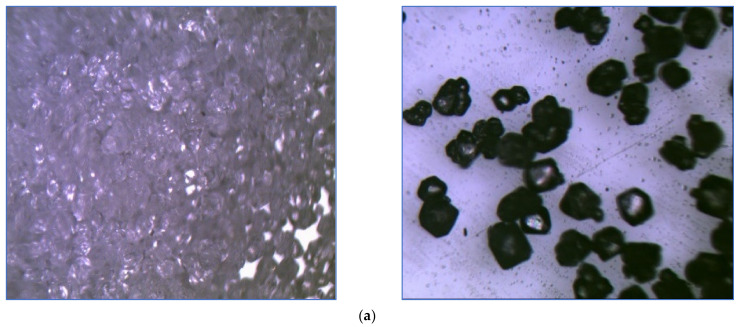

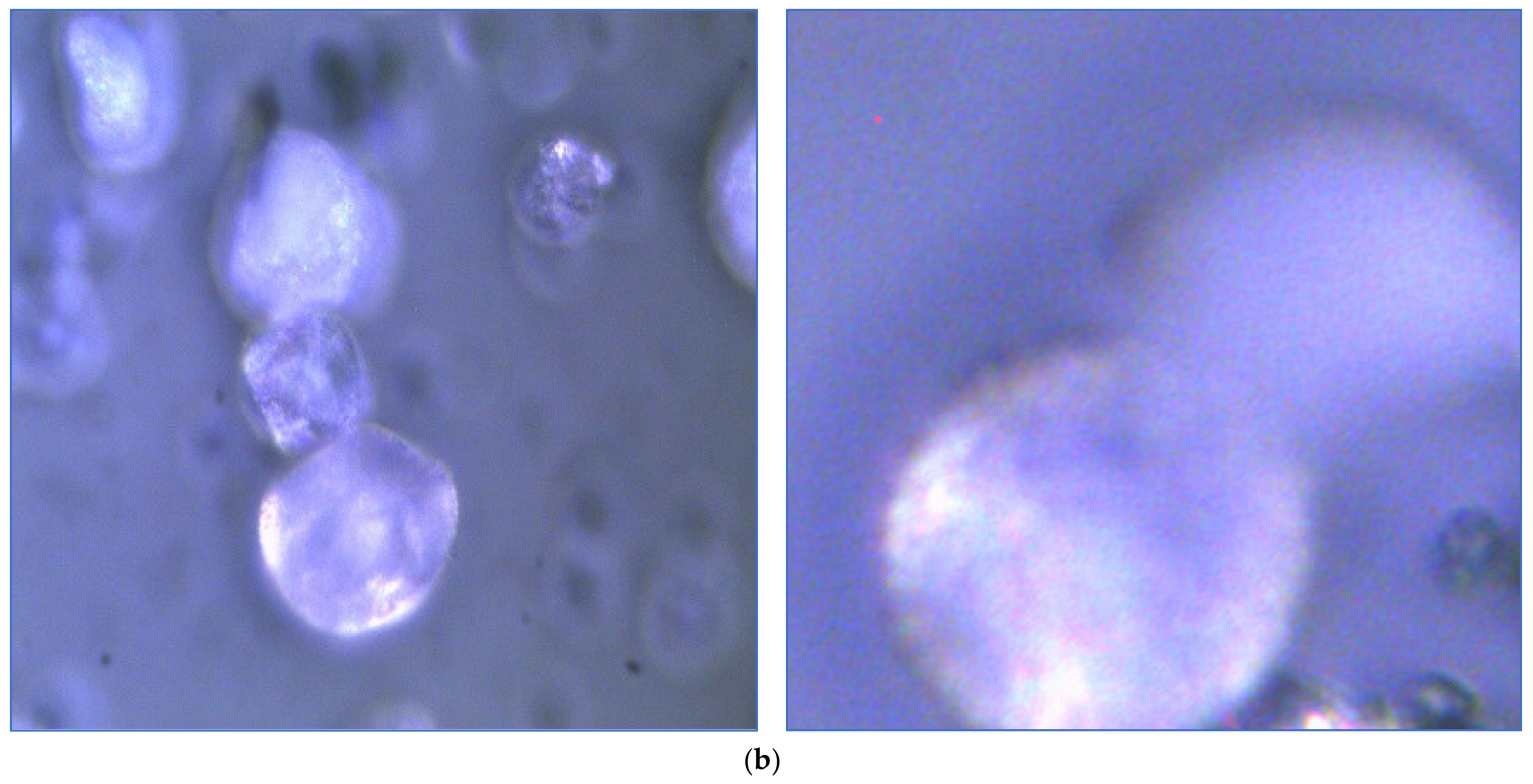
Metallurgical microscopic views of the morphology of particles of RDX: (**a**) before coating (20–50 times magnification); (**b**) after coating (100 times magnification).

The metallurgical microscopic views of RDX particles before and after coating are given in Figure 18.

### 3.8. Mass Flow Rate of RDX and NUPC-6 Samples

The results of mass flow rates of samples of RDX and NUPC-6 composition are tabulated in Table 1.

### 3.9. Bulk Density of RDX and NUPC-6 Samples

The results of bulk densities of samples of RDX and NUPC-6 composition are tabulated in Table 2.

### 3.10. Tapped Density of RDX and NUPC-6 Samples

The results of tapped densities of samples of RDX and NUPC-6 composition are tabulated in Table 3.

### 3.11. Compressibility Index, Hausner Ratio, and Flow Property of RDX and NUPC-6 Samples

The results of the compressibility index, Hausner ratio, and flow property of samples of RDX and NUPC-6 composition are tabulated in Table 4.

### 3.12. Velocity of Detonation NUPC-6, Polymer-Bonded Composition and Tetryl

The results of the velocity of detonation of three of each booster sample are given in Table 5.

## 4. Discussion

The present research work was conducted to develop a new polymer-bonded composition based on the solvent–slurry coating, which might be used as a booster and filler. Its sensitivity features are similar to the CH-6 composition; it is a new composition. It was optimized by process optimizations, i.e., the selection of n-hexane as a solvent for binder solution, gradation of RDX particles in the range of −100 to +150 mesh, and modification of polymer binder by adding diisooctylsebacate and SAE-10 oil. The composition of RDX was prepared using n-octane, toluene, and n-hexane as solvents. The boiling point ranges of n-octane, toluene, and n-hexane were 125–126 °C, 110–111 °C and 68–69 °C. It shows that n-hexane has a marginally good process safety range for distillation, and it also shows that the energy requirements for the distillation process are on the lower side as compared with other solvents. The higher the processing temperature for distillation and recovery of the solvent, the higher be risk involved in processing and vice versa.

Moreover, the toxicity of toluene and n-octane is on the higher side. The n-hexane, with a boiling point of 68–69 °C and the least toxicity of the three solvents used, was selected for the process optimized for NUPC-6 composition. The DSC thermograph of samples shows that the melting takes place from 17.42 min, and the liquid phase ends at 17.86 min, as shown by blue lines in Figure 7a. The maximum energy is released in the case of sample 1, at 20.95 min, and in the case of sample 2, at 21.10 min, as highlighted in black and red color lines in Figure 7a. The DSC thermographs of samples show that the melting range of both samples is from 204 to 206 °C, as highlighted by pink lines in Figure 7b. The melting of both samples was completed at 206 °C, and the maximum temperature rise in the case of sample 1 due to decomposition is 239.31 °C and 241.60 °C for sample 2, as highlighted by black and red lines, respectively. The results show that the graded RDX has good thermal stability [36]. The FTIR results of raw and modified polymer binders were investigated. The peaks at 3428.15 cm^−1^, 2327.69 cm^−1^, and 1616.48 cm^−1^ are thumbprints of polyisobutylene, as highlighted by purple lines [37]. The peaks at 3000–2800 cm^−1^, 1738.22 cm^−1^, 1461.10 cm^−1^, and 1171.97 cm^−1^ are thumbprints of diisooctylsebacate, as highlighted by blue lines [38]. The peaks at 805.15–711.44 cm^−1^ are thumbprints of SAE-10 lubricating oil, as highlighted by green lines [39]. The results are consistent with the quantity of added ingredients. Diisooctylsebacate and SAE-10 oil were added as an agent to reduce surface tension and reduce the viscosity of the polymer binder for coating. The XRD pattern of amorphous powder of sodium stearate in Figure 9a is a true representative of its nature. It has no sharp 2Ɵ plane angles [40].

Similarly, the XRD pattern of calcium stearate has the same trend in Figure 9b [41]. The results of peaks in XRD patterns show that both the stearates have fairly similar 2Ɵ plane angles without sharp peaks. The XRD pattern of graphite has a sharp 2Ɵ plane angle in the range 25–27 °C and 54–55 °C, confirming the granular form of the graphite, i.e., having sharp plane angles [42].

The AFM of the thin film of the raw polymer binder in Figure 10a shows the 200 nm × 200 nm area of the thin film. The maximum roughness of the thin film of raw polymer binder along the *z*-axis is 2.83 nm in Figure 11. The two-dimensional variation in the thickness of the thin film of raw polymer binder is from 0.080 nm to 0.720 nm in Figure 12. It shows that the ratio of the maximum to the minimum two-dimensional thickness variation is 9. The topography image of a thin film of raw polymer binder shows that the surface of raw polymer binder is very rough in Figure 13. The AFM of the thin film of the modified polymer binder in Figure 10b shows the 200 nm × 200 nm area of the thin film. The maximum roughness of the thin film of the modified polymer binder along the *z*-axis is 94.9 nm in Figure 14. The two-dimensional variation in the thickness of the thin film of modified polymer binder is from 10.4 nm to 11.4 nm in Figure 15. It shows that the ratio of the maximum to the minimum two-dimensional thickness variation is 1.1. The topography image of a thin film of modified polymer binder shows that the surface of modified polymer binder is very smooth in Figure 16. The results show that the modified polymer binder’s topography has lesser overall variation than the raw polymer binder’s topography.

The results of the SEM of the RDX sample show that it has orthorhombic morphology in Figure 17a. The morphology of the surface of the graded RDX sample shows that cracks have developed on the surface. These might have been induced due to decomposition based on exposure to high voltages during SEM, as shown in Figure 17b. The metallurgical microscopic views of RDX before coating and after coating were used to show the effect of coating RDX. The results show that the orthorhombic shape is changed to a spherical shape after coating and adding the ingredients in the new composition to a fair extent, as shown in Figure 18a and Figure 18b, respectively. The greater the degree of coating of the modified binder and mixing of ingredients, the greater the composition’s sphericity and vice versa.

The raw polymer binder with high weight average molecular mass is modified to reduce the batch preparation time and hardness. It increases the smoothness of the polymer binder. It increases the adhesive forces and increases the plasticity of the raw polymer binder. The addition of the polar and the plasticizer molecules of diisooctylsebacate and the viscosity-reducing agent, i.e., SAE-10 oil in a raw polymer binder, enhances the thin-film forming tendency of raw polymer binder. The dissolution time of the raw polymer binder with a high weight average molecular mass is 24 h in n-hexane at 45–50 °C with constant stirring on a hot plate. The dissolution time of the modified polymer binder is 2 h at 50 °C with constant stirring on a hot plate.

The addition of diisooctylsebacate increases intermolecular interactions between n-hexane and raw polymer binder with a high weight average molecular mass. The intermolecular interactions between matrix components of modified polyisobutylene cause the smoothness of the raw polymer binder. It reduces the tendency of forming large drops of solution of raw polymer binder with high weight average molecular mass while pouring in solution form with n-hexane for coating. Moreover, the powder composition prepared by coating raw polymer binder with high weight average molecular mass is rough due to larger variation in topography.

**Figure 19:**
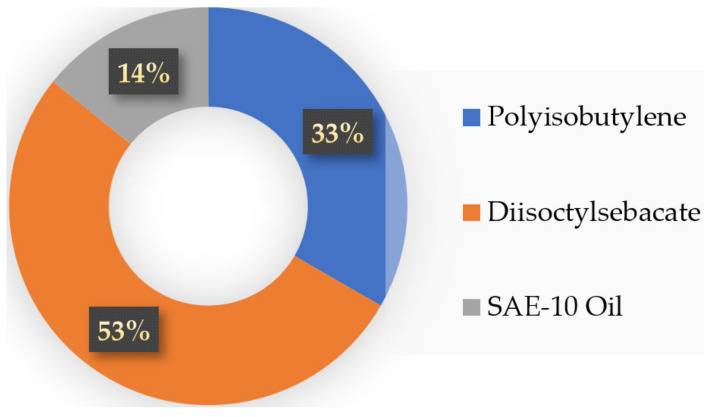
Composition of modified polymer binder.

The modified composition of the polymer binder consists of 33% polyisobutylene, 53% diisooctylsebacate, and 14% SAE-10 oil. The percent composition of the modified polymer binder is given in Figure 19.

The additives calcium stearate and graphite are added as dispersant and flow-enhancing agents, respectively. The dispersant avoided agglomeration of the polymer-coated particles of RDX with each other, and the flow-enhancing agent induced flowable characteristics in the composition even after adding low-density ingredients. The components of the NUPC-6 composition are 97.5% RDX, 0.17% polyisobutylene, 0.26% diisooctylsebacate, 0.07% SAE-10 oil, 1.5% calcium stearate, and 0.5% graphite.

**Figure 20:**
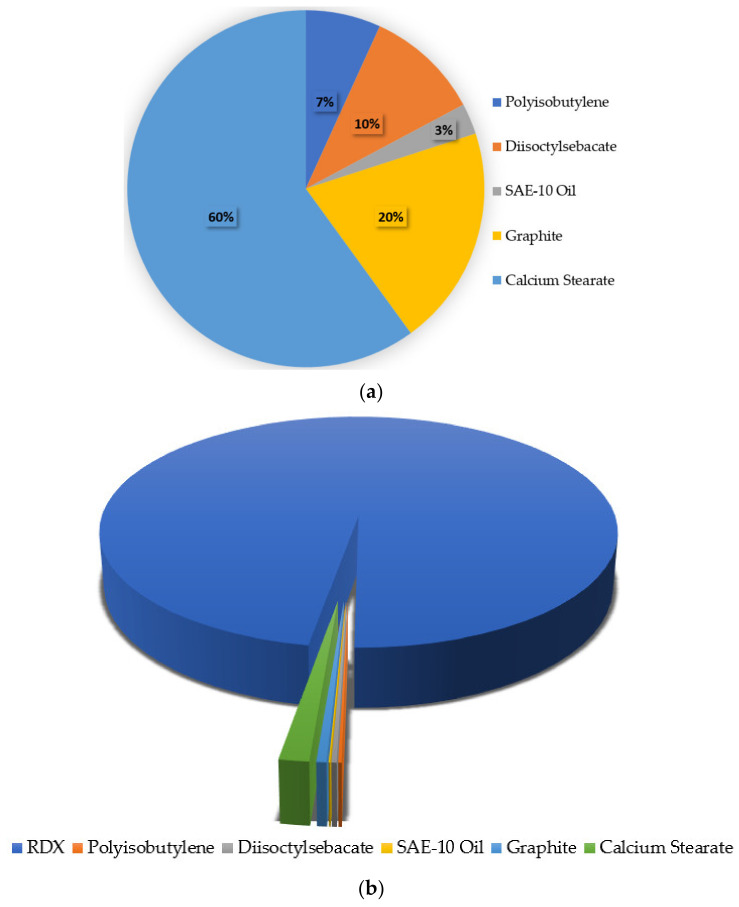
Pie chart of compositions of added ingredients and NUPC-6 composition: (**a**) composition of added ingredients to RDX (%); (**b**) composition of NUPC-6 (%).

The composition of added ingredients to RDX and of NUPC-6 are plotted in Figure 20. The calcium stearate being hydrophobic enables the use of the composition in under-water applications. The diisooctylsebacate, as a plasticizer, enables the extrusion of the composition. Graphite also enhances the flowability and phlegmatizes the composition. The greater the mass flow rate of the composition, the greater the flowing tendency of the composition and vice versa.

The comparison of the range of mass flow rates of RDX and the composition shows that the composition is flowable. The results show that the newly developed composition has good flowability using a filling funnel. The flow of composition is very important for its use as a booster and filler. The greater the flowability during filling, the greater the production rates and vice versa.

The mass flow rate, bulk or apparent density, and tapped density are the main features that characterize the bulk properties of powders. The average mass flow rate of RDX and the new composition is 63 g/s and 42 g/s, respectively, during filling operations using a filling funnel. The results of the mass flow rate show that the mass flow rate decreased due to the addition of low-density ingredients. The bulk or apparent density of powders is the characteristic that determines the actual volume allocated by a mass of loose powder that directly defines the design of filling dies and the range of the press motions required to pack and compact the loose powder. The average bulk or apparent density of samples in Table 2 is 0.66 g/cm^3^ and 0.50 g/cm^3^ for RDX and the new composition, respectively. The tapped density is a function of particle size distribution, particle shape, and surface roughness. It is always higher than the bulk or apparent density. The average tapped density of samples in Table 3 is 0.81 g/cm^3^ and 0.61 g/cm^3^. The results of the bulk or apparent and tapped density of RDX and the new composition show that the bulk or apparent density and tapped density dropped by a figure of 24% and 25%, respectively. It is due to the addition of low-density components like polyisobutylene, diisooctylsebacate, SAE-10 oil, and calcium stearate. The results are consistent with the nature of the process of coating and additives added in RDX. The compressibility index of a powder is a measure of its tendency to aggregate. It is a measure of the relative interparticulate interactions. A flowable powder has fewer inter-particle interactions, and the bulk and tapped densities become closer in value. For less flowing material, there are greater interparticle interactions that often result in lower bulk density and a greater difference between the bulk and tapped densities. These differences in particle interactions are reflected in the compressibility index. The average compressibility index of the RDX and the new composition samples in Table 4 is 18 and 17, respectively. The results show that the new composition is fairly flowable during filling operations based on the compressibility index. The Hausner ratio is closely related to the compressibility index.

The average Hausner ratio of the powder RDX and the new composition given in Table 4 is 1.22 and 1.21, respectively. The results again show that the new composition is fairly flowable during filling operations based on the range of the Hausner ratio [43]. The optimized composition is flowable during filling operations and might be used in automatic pelleting machines and filling machines. The VOD of the nitramine, Tetryl, and the new composition is measured and compared using detonators detonating the three booster samples of each via optical fiber technique with sensors. The sensors transmit the detonation initiation and end signals. The results show that the average velocity of detonation of the new composition and Tetryl is 8421 and 7273 m/s [44], respectively. The results show that the new composition NUPC-6 might replace Tetryl and CH-6 composition in applications in which greater plasticity as booster and filler is in high demand.

## 5. Conclusions

This research work was performed on the solvent–slurry coating to develop a flowable, polymer-bonded composition of RDX for use in boosters of large sizes and as fillers in linear-shaped charge extrusion. There are multiple steps and processes used in solvent–slurry coating. The mass flow rate, bulk density, tapped density, and Hausner ratio are the critical parameters for evaluating the flow property of the new powder composition of RDX. The results of flow property tests show that the composition is flowable during filling operations. The research work included the selection of solvent n-hexane, gradation of RDX from −100 to + 150 mesh, the modification of polymer binder, the addition of calcium stearate precipitates as a dispersant, and the addition of flow-enhancing agent graphite culminating into a new flowable, polymer-bonded composition. It was named NUPC-6 composition. The characterizations of the raw materials, modified materials, and composition were performed. The mass flow rate, bulk density, tapped density, compressibility index, Hausner’s ratio, and velocity of detonation of composition were also determined. The ibid shows that the NUPC-6 composition might be used as booster and filler applications demanding greater plasticity than Tetryl and CH-6 composition, i.e., as booster of greater sizes and filler for extrusion of lengthy linear-shaped charges. Its velocity of detonation is greater than the velocity of detonation of Tetryl.

## Figures and Tables

**Table 1:** Data of mass flow rate of RDX and NUPC-6.

Sample No.	Parameter	RDX		NUPC-6 Composition	
1	Mass (g)	200	±0.01	200	±0.01
	Time of Flow (s)	3	±0.01	5	±0.01
	Mass Flow Rate (g/s)	66.67	±0.22	40	±0.10
2	Mass (g)	200	±0.01	200	±0.01
	Time of Flow (s)	3	±0.01	5	±0.01
	Mass Flow Rate (g/s)	66.67	±0.22	40	±0.10
3	Mass (g)	200	±0.01	200	±0.01
	Time of Flow (s)	3	±0.01	4	±0.01
	Mass Flow Rate (g/s)	66.67	±0.22	50	±0.13
4	Mass (g)	200	±0.01	200	±0.01
	Time of Flow (s)	4	±0.01	5	±0.01
	Mass Flow Rate (g/s)	50	±0.12	40	±0.10
5	Mass (g)	200	±0.01	200	±0.01
	Time of Flow (s)	3	±0.01	5	±0.01
	Mass Flow Rate (g/s)	66.67	±0.22	40	±0.10

**Table 2:** Data of bulk densities of RDX and NUPC-6.

Sample No.	Parameter	RDX		NUPC-6 Composition	
1	Mass (g)	200	±0.01	200	±0.01
	Height of Heap (cm)	9.78	±0.01	10.72	±0.01
	Radius of Heap (cm)	5.4	±0.01	5.9	±0.01
	Bulk Volume of Heap (cm^3^)	298	±2.5	391	±2.5
	Bulk Density of Heap (g/cm^3^)	0.67	±0.008	0.51	±0.008
2	Mass (g)	200	±0.01	200	±0.01
	Height of Heap (cm)	9.82	±0.01	10.76	±0.01
	Radius of Heap (cm)	5.45	±0.01	5.92	±0.01
	Bulk Volume of Heap (cm^3^)	305	±2.5	395	±2.5
	Bulk Density of Heap (g/cm^3^)	0.66	±0.008	0.51	±0.008
3	Mass (g)	200	±0.01	200	±0.01
	Height of Heap (cm)	9.8	±0.01	10.74	±0.01
	Radius of Heap (cm)	5.44	±0.01	5.97	±0.01
	Bulk Volume of Heap (cm^3^)	303	±2.5	400	±2.5
	Bulk Density of Heap (g/cm^3^)	0.66	±0.008	0.5	±0.008
4	Mass (g)	200	±0.01	200	±0.01
	Height of Heap (cm)	9.82	±0.01	10.78	±0.01
	Radius of Heap (cm)	5.47	±0.01	6	±0.01
	Bulk Volume of Heap (cm^3^)	308	±2.5	406	±2.5
	Bulk Density of Heap (g/cm^3^)	0.65	±0.008	0.49	±0.008
5	Mass (g)	200	±0.01	200	±0.01
	Height of Heap (cm)	9.8	±0.01	10.74	±0.01
	Radius of Heap (cm)	5.44	±0.01	5.97	±0.01
	Bulk Volume of Heap (cm^3^)	303	±2.5	400	±2.5
	Bulk Density of Heap (g/cm^3^)	0.66	±0.008	0.5	±0.008

**Table 3:** Data of tapped densities of RDX and NUPC-6.

Sample No.	Parameter	RDX		NUPC-6 Composition	
1	Mass (g)	20	±0.01	20	±0.01
	Tapped Volume (cm^3^)	26	±0.01	34	±0.01
	Tapped Density (g/cm^3^)	0.77	±0.005	0.59	±0.005
2	Mass (g)	20	±0.01	20	±0.01
	Tapped Volume (cm^3^)	25	±0.01	33	±0.01
	Tapped Density (g/cm^3^)	0.8	±0.005	0.61	±0.005
3	Mass (g)	20	±0.01	20	±0.01
	Tapped Volume (cm^3^)	24	±0.01	33	±0.01
	Tapped Density (g/cm^3^)	0.83	±0.005	0.61	±0.005
4	Mass (g)	20	±0.01	20	±0.01
	Tapped Volume (cm^3^)	25	±0.01	32	±0.01
	Tapped Density (g/cm^3^)	0.8	±0.005	0.63	±0.005
5	Mass (g)	20	±0.01	20	±0.01
	Tapped Volume (cm^3^)	24	±0.01	34	±0.01
	Tapped Density (g/cm^3^)	0.83	±0.005	0.59	±0.005

**Table 4:** Data of compressibility index, Hausner ratio, and flow property of RDX and the composition.

Sample No.	Parameter	RDX		NUPC-6 Composition	
1	Bulk Density of Heap (g/cm^3^)	0.67	±0.008	0.51	±0.008
	Tapped Density (g/cm^3^)	0.77	±0.005	0.59	±0.005
	Compressibility Index	12.99	±1.61	13.56	±2.11
	Hausner’s Ratio	1.15	±0.02	1.16	±0.03
	Flow Property	Fairly Flowable		Fairly Flowable	
2	Bulk Density of Heap (g/cm^3^)	0.66	±0.008	0.51	±0.008
	Tapped Density (g/cm^3^)	0.8	±0.005	0.61	±0.005
	Compressibility Index	17.5	±1.61	16.39	±2.11
	Hausner’s Ratio	1.21	±0.02	1.20	±0.03
	Flow Property	Fairly Flowable		Fairly Flowable	
3	Bulk Density of Heap (g/cm^3^)	0.66	±0.008	0.5	±0.008
	Tapped Density (g/cm^3^)	0.83	±0.005	0.61	±0.005
	Compressibility Index	20.48	±1.61	18.03	±2.11
	Hausner’s Ratio	1.26	±0.02	1.22	±0.03
	Flow Property	Fairly Flowable		Fairly Flowable	
4	Bulk Density of Heap (g/cm^3^)	0.65	±0.008	0.49	±0.008
	Tapped Density (g/cm^3^)	0.8	±0.005	0.63	±0.005
	Compressibility Index	18.75	±1.61	22.22	±2.11
	Hausner’s Ratio	1.23	±0.02	1.29	±0.03
	Flow Property	Fairly Flowable		Fairly Flowable	
5	Bulk Density of Heap (g/cm^3^)	0.66	±0.008	0.5	±0.008
	Tapped Density (g/cm^3^)	0.83	±0.005	0.59	±0.005
	Compressibility Index	20.48	±1.61	15.25	±2.11
	Hausner’s Ratio	1.26	±0.02	1.18	±0.03
	Flow Property	Fairly Flowable		Fairly Flowable	

**Table 5:** Data of velocity of detonation of NUPC-6 composition and Tetryl.

Sample No.	Specifications	RDX		Tetryl	
1	Mass of Booster (g)	18.1	±0.01	18	±0.01
	Diameter of Booster (cm)	2.98	±0.01	2.98	±0.01
	Height of Booster (cm)	1.6	±0.01	1.6	±0.01
	Volume of Booster (cm^3^)	11.15	±0.15	11.08	±0.15
	Density of Booster (g/cm^3^)	1.62	±0.02	1.62	±0.02
	Distance Between Probes (m)	0.016	±0.0001	0.016	±0.0001
	Time Recorded (s)	1.9 × 10^−6^	±0.1 × 10^−6^	2.2 × 10^−6^	±0.1 × 10^−6^
	Velocity of Detonation (m/s)	8421	±60	7273	±60
2	Mass of Booster (g)	18	±0.01	18	±0.01
	Diameter of Booster (cm)	2.98	±0.01	2.98	±0.01
	Height of Booster (cm)	1.59	±0.01	1.59	±0.01
	Volume of Booster (cm^3^)	11.08	±0.15	11.08	±0.15
	Density of Booster (g/cm^3^)	1.62	±0.02	1.62	±0.02
	Distance Between Probes (m)	0.0159	±0.0001	0.0159	±0.0001
	Time Recorded (s)	1.9 × 10^−6^	±0.1 × 10^−6^	2.2 × 10^−6^	±0.1 × 10^−6^
	Velocity of Detonation (m/s)	8368	±60	7227	±60
3	Mass of Booster (g)	18.2	±0.01	18.1	±0.01
	Diameter of Booster (cm)	2.98	±0.01	2.98	±0.01
	Height of Booster (cm)	1.61	±0.01	1.61	±0.01
	Volume of Booster (cm^3^)	11.22	±0.15	11.22	±0.15
	Density of Booster (g/cm^3^)	1.62	±0.02	1.61	±0.02
	Distance Between Probes (m)	0.0161	±0.0001	0.0161	±0.0001
	Time Recorded (s)	1.9 × 10^−6^	±0.1 × 10^−6^	2.2 × 10^−6^	±0.1 × 10^−6^
	Velocity of Detonation (m/s)	8474	±60	7318	±60

## Data Availability

Data are contained within the article.
